# Cost-minimization analysis of three decision strategies for cardiac revascularization: results of the “suspected CAD” cohort of the european cardiovascular magnetic resonance registry

**DOI:** 10.1186/s12968-015-0222-1

**Published:** 2016-01-11

**Authors:** Karine Moschetti, Steffen E. Petersen, Guenter Pilz, Raymond Y. Kwong, Jean-Blaise Wasserfallen, Massimo Lombardi, Grigorios Korosoglou, Albert C. Van Rossum, Oliver Bruder, Heiko Mahrholdt, Juerg Schwitter

**Affiliations:** 1 Healthcare Evaluation Unit, Institute of Social and Preventive Medicine (IUMSP), Lausanne, Switzerland; 2 Technology Assessment Unit, University Hospital of Lausanne, Lausanne, Switzerland; 3 William Harvey Research Institute, NIHR Cardiovascular Biomedical Research Unit at Barts, Queen Mary University of London, London, UK; 4 Clinic Agatharied, Academic Teaching Hospital, University of Munich, Munich, Germany; 5 Brigham and Women’s Hospital, Harvard Medical School, Boston, USA; 6 Policlinics of San Donato, Italian Research Hospital, Milano, Italy; 7 University Hospital of Heidelberg, Heidelberg, Germany; 8 VU University Medical Center, Amsterdam, The Netherlands; 9 Elisabeth Hospital, Department of Cardiology and Angiology, Elisabeth Hospital Essen, Essen, Germany; 10 Department of Cardiology, Robert Bosch Hospital, Stuttgart, Germany; 11 Division of Cardiology, Director Cardiac MR Center, University Hospital Lausanne - CHUV, Rue du Bugnon 46, 1011 Lausanne, Switzerland

## Abstract

**Background:**

Coronary artery disease (CAD) continues to be one of the top public health burden. Perfusion cardiovascular magnetic resonance (CMR) is generally accepted to detect CAD, while data on its cost effectiveness are scarce. Therefore, the goal of the study was to compare the costs of a CMR-guided strategy vs two invasive strategies in a large CMR registry.

**Methods:**

In 3’647 patients with suspected CAD of the EuroCMR-registry (59 centers/18 countries) costs were calculated for diagnostic examinations (CMR, X-ray coronary angiography (CXA) with/without FFR), revascularizations, and complications during a 1-year follow-up. Patients with ischemia-positive CMR underwent an invasive CXA and revascularization at the discretion of the treating physician (=CMR + CXA-strategy). In the hypothetical invasive arm, costs were calculated for an initial CXA and a FFR in vessels with ≥50 % stenoses (=CXA + FFR-strategy) and the same proportion of revascularizations and complications were applied as in the CMR + CXA-strategy. In the CXA-only strategy, costs included those for CXA and for revascularizations of all ≥50 % stenoses. To calculate the proportion of patients with ≥50 % stenoses, the stenosis-FFR relationship from the literature was used. Costs of the three strategies were determined based on a third payer perspective in 4 healthcare systems.

**Results:**

Revascularizations were performed in 6.2 %, 4.5 %, and 12.9 % of all patients, patients with atypical chest pain (*n* = 1’786), and typical angina (*n* = 582), respectively; whereas complications (=all-cause death and non-fatal infarction) occurred in 1.3 %, 1.1 %, and 1.5 %, respectively. The CMR + CXA-strategy reduced costs by 14 %, 34 %, 27 %, and 24 % in the German, UK, Swiss, and US context, respectively, when compared to the CXA + FFR-strategy; and by 59 %, 52 %, 61 % and 71 %, respectively, versus the CXA-only strategy. In patients with typical angina, cost savings by CMR + CXA vs CXA + FFR were minimal in the German (2.3 %), intermediate in the US and Swiss (11.6 % and 12.8 %, respectively), and remained substantial in the UK (18.9 %) systems. Sensitivity analyses proved the robustness of results.

**Conclusions:**

A CMR + CXA-strategy for patients with suspected CAD provides substantial cost reduction compared to a hypothetical CXA + FFR-strategy in patients with low to intermediate disease prevalence. However, in the subgroup of patients with typical angina, cost savings were only minimal to moderate.

**Electronic supplementary material:**

The online version of this article (doi:10.1186/s12968-015-0222-1) contains supplementary material, which is available to authorized users.

## Background

Coronary artery disease (CAD) continues to be a major source of public health burden particularly in industrialized countries [1]. For the European Union, the estimated costs for CAD management were 60 billion Euros in 2009, of which approximately 20 billion Euros were attributed to direct health care costs [2]. Similarly, the total direct costs of CAD in the United States were estimated to be 107 billion dollars in the same time period [3]. Patients with myocardial ischemia benefit most from revascularizations, as the presence of myocardial ischemia is a strong predictor of major adverse cardiovascular outcomes. Accordingly, current guidelines recommend revascularizing patients with stable CAD if substantial myocardial ischemia is confirmed by either non-invasive ischemia testing or fractional flow reserve (FFR) [4–7]. Fearon and coworkers demonstrated that such an FFR-, i.e. ischemia-guided approach, was not only safe and effective in improving patient outcomes, but reduced costs during the first year after percutaneous coronary interventions (PCI) comparing to a luminal anatomy-guided approach [8]. Invasive CXA, particularly when combined with FFR, is an alternative to non-invasive testing and should be considered in intermediate to high risk patients, i.e. with an annual mortality ≥1 % according to ESC guidelines [5]. The AHA/ACC guidelines on ischemic heart disease justify the use of CXA as first line test to define the extent and severity of CAD in patients with a high likelihood of severe disease (based on clinical assessment and/or exercise ECG testing) [9]. Cardiovascular magnetic resonance (CMR) is now well established as a reliable and safe technique to evaluate ischemia in patients with known or suspected CAD [10–16] and it is recommended as a class I or IIa test in European and US guidelines for stable CAD, respectively [4–7]. However, few data are available to estimate the potential cost savings using the CMR-based approach [17].

Assessment of patients with suspected CAD aiming at effective clinical decision-making needs to not only consider patient factors including cardiovascular risk factor profile, presenting sign and symptoms, but the presence of myocardial ischemia and the coronary arterial anatomy. The aim of this study was to compare the costs of a CMR-guided strategy vs two invasive strategies. The costs of these strategies were assessed from a health care payer perspective for the German, United Kingdom, Swiss, and United States health care systems.

## Methods

### Definitions of strategies

**Fig. 1:**
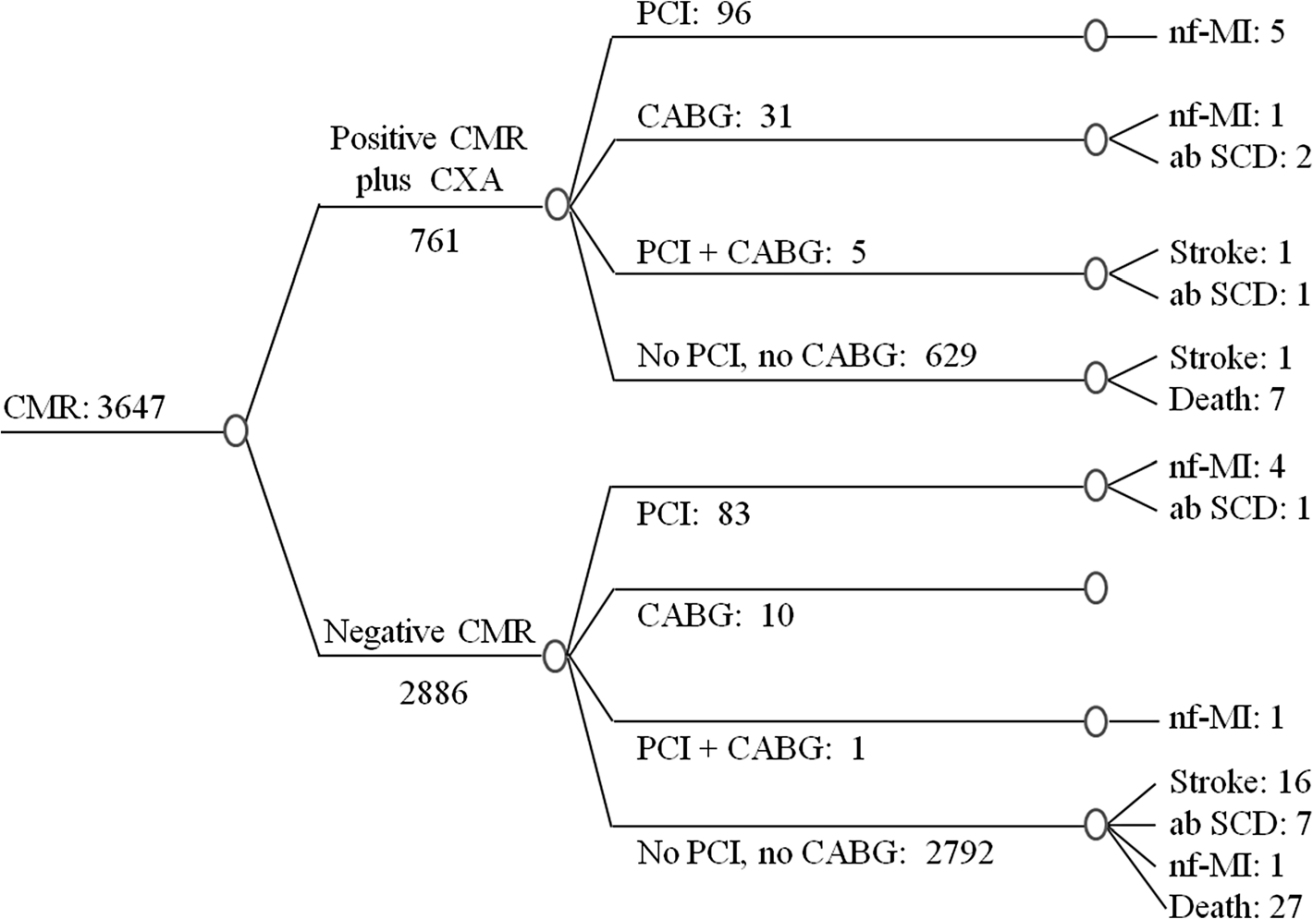
Decision tree and outcome in the study population – CMR + CXA strategy. Diagnostic pathway, treatment, and outcomes are shown for the CMR + CXA strategy in the 3’647 patients of the European CMR Registry. nf-MI: non-fatal MI; ab SCD: aborted SCD

**Fig. 2:**
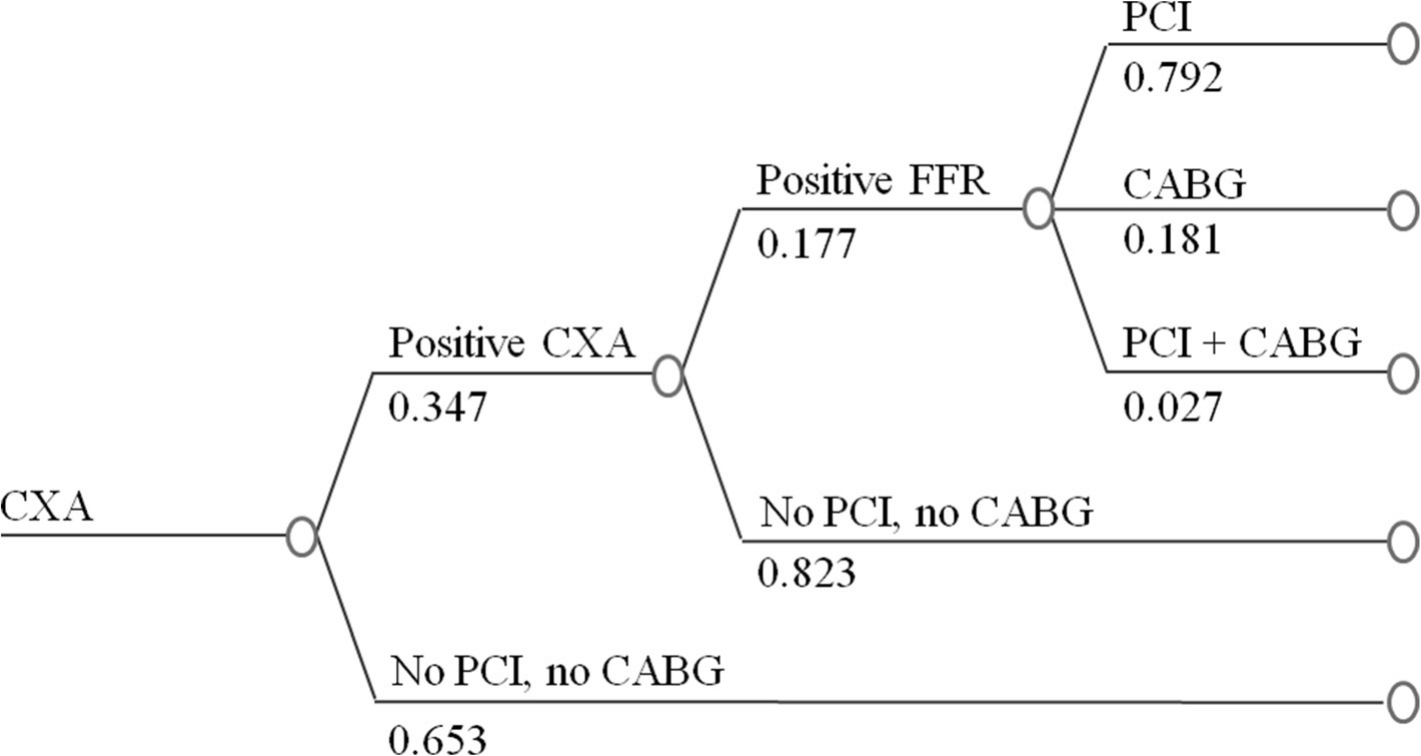
The CXA + FRR guided strategy. A hypothetical invasive CXA + FFR strategy is applied to the patients of the European CMR Registry

**Fig. 3:**
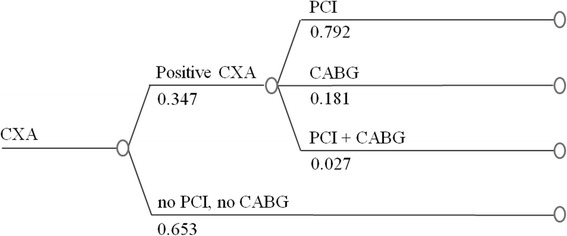
The CXA-only strategy. With this hypothetical strategy, anatomy as defined by invasive x-ray coronary angiography is the only test for decision making, no ischemia testing is used. Revascularizations are performed in patients with ≥50 % coronary stenosis

In the CMR-based strategy (CMR + CXA, Fig. 1), only patients positive for ischemia on CMR were referred to a CXA examination with potential revascularization performed at the discretion of the treating physician. For the CMR + CXA strategy costs included those for the initial CMR, for the CXA in the ischemia-positive patients, and for revascularizations and complications (as shown in Fig. 1). For details on cost calculations of the strategies, see also section below (costs of the different procedures and strategies). For comparison, a hypothetical CXA + FFR strategy was designed which starts with a CXA examination and in case of ≥50 % stenosis in a coronary artery, an FFR testing is added. In the registry population the revascularization rate was 6.2 % and we assumed FFR to be positive with the same proportion in the CXA + FFR strategy. Extrapolating from the correlation between FFR and diameter stenosis from the literature [18, 19], and assuming 6.2 % positive FFR tests (FFR ≤ 0.80) we calculate that approximately 35 % coronary arteries have ≥50 % diameter stenosis (for formulas see Additional file 1). Accordingly, 35 % of patients were assumed to undergo FFR testing to yield 6.2 % ischemia positive findings (Fig. 2). We applied the annual hard event rate observed from the European CMR registry, 0.38 % annually of cardiac death and non-fatal MI, to both the CMR + CXA and CXA + FFR strategies. This assumption is supported by strong evidence that both, FFR [20–22] and CMR [23–25] provide accurate prognostic information at a similar level. We also calculated the costs for a third CXA-only strategy (Fig. 3) which includes costs for the initial CXA and those for revascularizations in all patients with ≥50 % coronary stenoses as described by Moschetti and coworkers [19]. Finally, these calculations were also performed for the 2 sub-groups of patients with typical angina and atypical chest pain.

### Patient population

Data from the European CMR registry were used for this analysis [24]. The prospective “Suspected CAD” cohort aims to assess the prognostic value of CMR in a clinical routine setting by collecting data on subsequent treatment and major adverse cardiac events (MACE) during a follow-up of 1 year after the CMR examination [26]. The primary combined end-point of MACE included all-cause death, aborted sudden cardiac death (SCD), non-fatal MI, and stroke. The protocol was approved by the ethics committee at each participating institution and all study participants provided written informed consent. The present analysis includes 3’647 patients with suspected CAD who underwent CMR for ischemia testing and for whom the one-year follow-up was completed (Table 1). For ischemia testing, a first-pass perfusion approach was used [10–14] with vasodilation induced by adenosine (dipyridamole was used in one center; *n* = 10). Patients were classified as ischemia-positive, if ≥1 segment was ischemic by visual reading (using a 16-segment model) [26].

**Table 1:** Baseline characteristics

	Total population	Atypical chest pain	Typical angina	*p*-values ^a^
Demographics				
n (%)	3’647 (100 %)	1’786 (49.0 %)	582 (16.0 %)	-
Male (%)^†^	58.7 %	45.7 %	42.8 %	<0.001
Age at baseline (y); mean (range) ^‡^	61.6 (14–92)	61.1 (14–92)	62.6 (26–88)	<0.05
Weight (kg); mean (range) ^§^	80.4 (28–183)	80.2 (30–183)	79.7 (28–182)	ns
Risk profile				
Hypertension ^§^				ns
- none	38.1 %	39.1 %	35.4 %	
- treated	58.1 %	56.7 %	60.3 %	
- untreated	3.9 %	4.3 %	4.3 %	
Dyslipidemia ^ǁ^	42.2 %	40.5 %	45.9 %	0.059
Diabetes mellitus ^ǁ^	13.3 %	10.8 %	15.0 %	<0.001
Smoker ^ǁ^				ns
- No	74.5 %	73.9 %	73.9 %	
- Current	12.9 %	12.9 %	13.8 %	
- Previous	12.7 %	13.2 %	12.4 %	
Family history of CAD ^§^	27.0 %	28.3 %	29.4 %	<0.05
Reasons for CAD work-up				
- Patient complaints	72.7 %	89.7 %	89.7 %	<0.001
- Presence of cardiovascular risk factors	55.9 %	53.4 %	53.1 %	<0.001
- Ambiguous Stress ECG	20.2 %	17.4 %	14.4 %	<0.001
- Ambiguous Stress Echocardiography	1.9 %	1.4 %	1.2 %	<0.01
- Ambiguous Stress SPECT	0.3 %	0.2 %	0.5 %	ns
- Ambiguous Cardiac CT	0.9 %	0.7 %	0.5 %	ns
Treatment: n (%)				
Revascularizations	226 (6.2 %)	81 (4.5 %)	75 (12.9 %)	<0.001
- PCI only	179 (4.9 %)	70 (3.9 %)	53 (9.1 %)	<0.001
- CABG only	41 (1.1 %)	10 (0.6 %)	19 (3.3 %)	<0.001
- PCI and CABG	6 (0.2 %)	1 (0.1 %)	3 (0.5 %)	0.059
Outcome (complications): n (%)				
Primary endpoint	75 (2.1 %)	33 (1.9 %)	11 (1.9 %)	ns
- Mortality : all cause	34 (0.9 %)	15 (0.8 %)	7 (1.2 %)	ns
- Cardiac death	7 (0.2 %)	6 (0.4 %)	0 (0.0 %)	ns
- Cardiac death and unknown cause	23 (0.6 %)	13 (0.7 %)	2 (0.3 %)	ns
- Non-fatal myocardial infarction	11 (0.3 %)	5 (0.2 %)	2 (0.3 %)	ns
- Aborted sudden cardiac death	8 (0.3 %)	4 (0.2 %)	1 (0.2 %)	ns
- Stroke	18 (0.5 %)	10 (0.6 %)	1 (0.2 %)	ns

^a^ Differences for age and weight were assessed by one-way ANOVA and for the other parameters by the Chi-square statistic. *P*-values >0.10 are reported as ns. Reasons for CAD work-up may add up to >100 % as several reasons per patient may apply. ^†^ n = 3’643; ^‡^ n = 3’646; ^§^ n = 3’642; ^ǁ^ n = 3’641

### Costs of the different procedures and strategies

The analysis was performed from a health care payer perspective using 2014 unit costs data in Euros (€) for Germany, in pounds (£) for the United Kingdom, in Swiss Francs (CHF) for Switzerland, and in US Dollars (US$) for the United States (for details on health care systems, see reference [19]). We used reimbursement rates (tariffs) to assess the costs of procedures. See Additional file 2 for details on the sources of information used to derive the costs of the different tests in every country.

The average costs per patient for the 3 strategies were calculated by multiplying the proportion of patients in the different branches of the diagram (Figs 1, 2 and 3) with the unit costs of the different tests, revascularizations, and/or treatments of complications (Table B1 in Additional file 2).

In patients with revascularizations, the costs of one-year treatment with clopidogrel and aspirin were included. By contrast, costs of drugs associated with the management of risk factors were not taken into account, as risk factors should be treated in all patients irrespective of the presence or absence of ischemia. Finally, death was not associated with any costs. Given the time horizon of the analysis of 12 months, no discount rate was applied.

### Sensitivity analysis

To assess the influence of various cost parameters on the results, one-way deterministic sensitivity analyses were performed where input parameters were varied one at a time while the remaining values were held at their baseline values. Thus, the model was re-run with changes in the costs of the diagnostic tests of CMR, FFR, and CXA. As the CMR + CXA strategy and the CXA + FFR strategy were assumed to yield the same proportion of ischemia-positive patients, the revascularization procedures would not differ for the 2 arms. Accordingly, costs for treatment were not varied. As costs for the various tests may differ considerably in various geographical regions of the 4 countries and as costs for FFR testing are not (yet) well defined in all 4 health care systems, a break-even analysis was also performed to illustrate the magnitude of reimbursement changes needed to result in equal costs for the 3 strategies.

### Statistics

Categorical data are reported as frequencies and continuous data as mean ± SD. Differences between patient groups were assessed using one-way ANOVA and Chi-square statistics where appropriate (Table 1). A *p*-value <0.05 was considered statistically significant.

## Results

### Demographics

Based on CMR testing, 20.9 % of patients were diagnosed with ischemia and 17.4 % of these patients were revascularized (72.7 % by PCI, 23.5 % by CABG, and 3.8 % underwent both, PCI and CABG). Additionally, 3.3 % of the CMR-negative patients were revascularized. In the sub-group of patients with typical angina, ischemia was diagnosed by CMR in 34.9 % and revascularizations were performed in 23.2 % of these patients. For outcomes, see Table 1. During the CMR examination, no major complications occurred (for details, see Additional file 3, Table C).

### Cost analysis

**Fig. 4:**
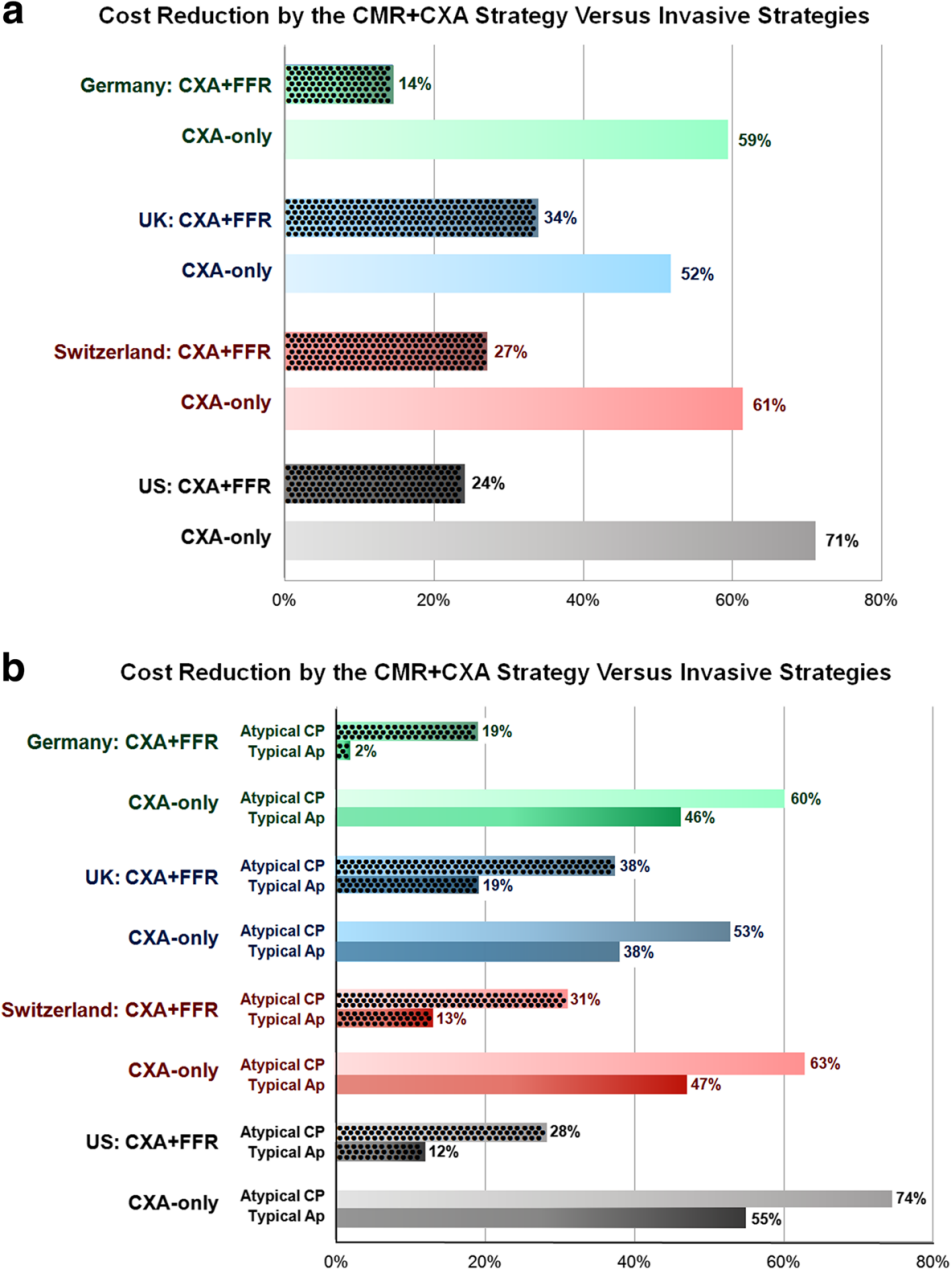
Percentage of cost reductions of the CMR + CXA strategy in comparison to the CXA + FFR and CXA-only strategies for the German, UK, Swiss, and US health care systems. 4**a** Cost reductions for the CMR + CXA strategy in the entire study population of 3’647 patients. 4**b** Cost reductions for the CMR + CXA strategy in the subgroups of patients with atypical chest pain (*n* = 1’786) and with typical angina (*n* = 582)

The average costs per patient for the 3 strategies in the 4 countries with all diagnostic tests (CMR and CXA with/without FFR) performed as outpatient procedures are given in Table 2. The cost reductions by the CMR + CXA strategy in the 4 countries are summarized in Fig. 4a. Costs reductions of CMR + CXA vs CXA + FFR were highest in the UK (34 %) and lowest in Germany (14 %), with US and Switzerland positioned in between with reductions of 24 % and 27 %, respectively.

**Table 2:** Costs of the 3 strategies per health care system (*n* = 3’647)

	Costs CMR + CXA	Costs CXA + FFR	Costs CXA-only	% Cost reduction of CMR + CXA versus CXA + FFR	% Cost reduction of CMR + CXA versus CXA-only	% Cost reduction of CXA + FFR versus CXA-only
German context (€)						
Main analysis (*n* = 3'647)	932	1'090	2'298	14.5	59.4	52.6
Main analysis (*n* = 3'647) without rehab. & without cardiologist's visit	919	1'082	2'290	15.1	59.9	52.8
- Atypical chest pain (*n* = 1'786)	787	971	1'990	19.0	60.5	51.2
- Atypical chest pain (*n* = 1'786) without rehab. & without cardiologist's visit	780	966	1'985	19.3	60.7	51.3
- Typical angina pectoris (*n* = 582)	1'466	1'500	2'690	2.3	45.5	44.2
- Typical angina pectoris (*n* = 582) without rehab. & without cardiologist's visit	1'456	1'514	2'704	3.8	46.2	44.0
UK context (£)						
Main analysis (*n* = 3'647)	1'075	1'623	2'224	33.8	51.7	27.0
- Atypical chest pain (n = 1'786)	968	1'552	2'052	37.6	52.8	24.4
- Typical angina pectoris (*n* = 582)	1'513	1'866	2'444	18.9	38.1	23.7
Swiss context (CHF)						
Main analysis (*n* = 3'647)	3'252	4'451	8'399	26.9	61.3	47.0
Main analysis (*n* = 3'647) without rehab. & without cardiologist's visit	3'191	4'420	8'368	27.8	61.9	47.2
- Atypical chest pain (*n* = 1'786)	2'783	4'044	7'520	31.2	63.0	46.2
- Atypical chest pain (*n* = 1'786) without rehab. & without cardiologist's visit	2'733	4'017	7'493	32.0	63.5	46.4
- Typical angina pectoris (*n* = 582)	5'074	5'816	9'511	12.8	46.7	38.9
- Typical angina pectoris (*n* = 582) without rehab. & without cardiologist's visit	4'983	5'784	9'479	13.8	47.4	39.0
US context ($)						
Main analysis (*n* = 3'647)	1'740	2'292	6'022	24.1	71.1	61.9
Main analysis (*n* = 3'647) with cardiologist's visit	1'759	2'294	6'024	23.3	70.8	61.9
- Atypical chest pain (*n* = 1'786)	1'429	1'996	5'588	28.4	74.4	64.3
- Atypical chest pain (*n* = 1'786) with cardiologist's visit	1'444	1'997	5'589	27.7	74.2	64.3
- Typical angina pectoris (*n* = 582)	2'947	3'335	6'592	11.6	55.3	49.4
- Typical angina pectoris (*n* = 582) with cardiologist's visit	2'983	3'336	6'593	10.6	54.8	49.4

As expected, in the study population with typical angina, the rate of revascularizations was higher than in the total population (Table 1). Also, in the population with typical angina, the CMR + CXA approach yielded lower cost savings vs the CXA + FFR strategy ranging from 2.3 % for Germany to 18.9 % for UK and with cost savings for US and Switzerland in between with 11.6 % and 12.8 %, respectively (see Table 2 and Fig. 4b). Cost savings in the population with atypical chest pain symptoms were comparable to the entire study population (see Table 2 and Fig. 4b).

Inclusion or exclusion of costs for rehabilitation after a non-fatal MI (for Germany and Switzerland) or of costs for a visit to see a cardiologist in the first year after revascularization (for Germany, Switzerland, and US) did not significantly influence the differences between strategies (difference of <1 percentage point, Table 2).

### Sensitivity analysis

Results of the sensitivity analyses are given in the Additional file 4 (Tables D1-D4). Generally, a 10 % change in the costs of a diagnostic test (changes introduced into the model one by one) leads to variations ranging from 1 % to 8 % in final costs of the 3 strategies. A 10 % increase in the CMR costs leads to a reduction of 0.7 to 4.3 percentage points on the cost savings of the CMR + CXA strategy vs CXA + FFR in the 4 health care systems. A 10 % decrease in the CXA costs would reduce the cost savings of the CMR + CXA strategy vs CXA + FFR by 2.4 to 4.5 percentage points.

When varying the proportion of patients which undergo FFR in the CXA + FFR strategy from 30 % to 55 %, the sensitivity analysis yields changes in costs in favor of the CMR + CXA strategy for all health care systems and all populations studied with the only exception for the patients with typical angina in the German system, where costs savings were minor ranging from 0.8 % to 6.1 % (Additional file 4, Tables D1-D4).

## Discussion

### Cost minimization by the CMR-guided strategy to manage patients with suspected CAD

Our study represents an effort of cost-benefits analysis across systems from 4 countries extrapolated from “real-world” multinational data from the European CMR registry. Using the CMR + CXA strategy cost savings ranging from 14 % to 34 % can be expected compared to an invasive CXA + FFR strategy when applied on a population of low to intermediate prevalence of disease. By the same token, substantial cost savings ranging from 19 % to 38 % can be anticipated in the patients with atypical chest pain. However, in the population with typical angina, cost savings were minimal in the German health care system and only moderate in the US and Swiss systems with 12 %-13 %, while they remained substantial in the UK with 19 % cost savings. This sub-population analysis underscores the importance of pre-test probabilities when searching for the most cost-effective work-up strategy. Thus, in a low pre-test probability population, which dominates those referred to non-invasive testing, cost savings might be substantial with a CMR + CXA strategy, even if the costs of an invasive CXA is added to all patients with a positive CMR. With an increasing pre-test probability, as e.g. in the typical angina population, substantial cost savings may persist in some countries but may decline in others.

The potential cost savings were observed if all interventions were calculated as out-patient procedures and by taking into account the costs for revascularizations and complications during the first year after PCI and/or CABG. This favorable cost profile of the CMR-based strategy in a population with low to intermediate disease prevalence is in line with a cost-effectiveness analysis performed in the setting of the CE-MARC trial [27]. CMR as a first line test followed by CXA in patients with a positive or inconclusive CMR study was found cost-effective in this population at the higher end of the National Institute for Health and Clinical Excellence (NICE) threshold range [27]. Of note, the European CMR registry data are collected from a network of 59 centers representing 18 countries and are therefore highly likely to reflect broad CMR performance achievable in current routine cardiology practice. This study design also accounted for costs of mis-classifications (i.e. for false negative CMR studies), as costs of invasive tests, revascularizations, and costs for complication management in CMR-negative patients (=false negatives) were added to the overall costs of the CMR + CXA strategy. Interestingly, European and US guidelines recommend to consider CXA as a first test, if the annual mortality is relatively high, i.e. ≥1 % [5], and/or if results of noninvasive testing (exclusive of stress imaging) indicate a high likelihood of CAD (as e.g. in long-standing diabetes or in patients with electrocardiography with diffuse ischemic changes in multiple territories) [9]. The presented results indicate, that current guidelines on invasive CXA utilization in stable CAD [5, 9] are also valid when economic aspects are taken into account as cost savings of the CMR + CXA strategy were relatively small (or almost absent) in the studied population with typical angina and an annual mortality >1 %.

If the CMR + CXA strategy is compared with a CXA-only strategy, cost savings can be as high as 52–71 %, most likely due to the fact that ischemia testing reduces the need for revascularizations. Similar results have been shown in the past in prospective trials comparing a combined CXA and FFR approach vs a CXA-only approach [8, 28]. In a simulation, Moschetti et al. included the FFR ischemia testing in the model and showed that a CMR-based strategy was more cost-effective than CXA combined with FFR when applied to a population of low to intermediate pretest likelihood of CAD [19]. The results of the current study derived from a real patient population with an ischemia prevalence of 21 % now confirm these model simulations of Moschetti [19], which predicted cost-effectiveness in the German, UK, Swiss, and US health care systems in populations with an ischemia prevalence below 62 % to 83 %.

The European CMR registry design requires indications for CMR to be in accordance with appropriate use criteria established by recognized professional organizations [26, 29]. Interestingly, the prevalence of ischemia on stress perfusion CMR in a population fulfilling the appropriate use criteria was 18.8 % (vs 4.8 % in the rarely appropriate group) [30], which is close to the prevalence of 20.9 % in the current study. This finding may indirectly support the notion that indications in the registry were following current appropriate use criteria in most cases.

### Prognostic power of stress perfusion CMR in a real-world multi-center setting

Since false negative tests are likely to decrease quality of life and to increase costs by the management of complications of unrecognized disease, it is important to assess the prognostic performance of non-invasive methods. In this large unselected patient population with suspected CAD, a normal perfusion-CMR predicted an excellent outcome with an annual event rate for cardiac death and non-fatal MI of 0.38 %, which increased to 1.11 %, if deaths of unknown cause and aborted SCD were added. These registry outcome data match well with previous prospective CMR single center studies reporting annual event rates of cardiac death and non-fatal MI in ischemia-negative patients of 0.7 % per year [23, 25].

In the CMR negative patients revascularizations occurred in 3.3 % and in the sub-group with typical angina, they occurred in 7.4 %, which might represent false negative CMR examinations. However, no FFR proof was required to guide these revascularizations of CMR negative patients. In addition, in the FFR-negative population of FAME 2, a similar revascularization rate was observed with 12.0 % over 2 years [22]. It might be speculated that progression of disease over 1 year post-testing in ischemia-negative patients could partly account for these revascularizations. On the other hand, only 17.4 % of the CMR-positive patients were revascularized and only 23.2 % of the patients with typical angina. This is most likely explained by the fact, that patients by definition were categorized as “ischemic” with at least one segment positive on CMR [26], while it is recommended to revascularize patients with 2 or more ischemic segments [5].

### Limitations

The costs in the 2 invasive strategies were calculated based on the relationship between the stenosis degree and FFR-positive findings as reported in the literature [18, 19]. This relationship was not verified in the study population. Sensitivity analyses, however, demonstrated cost savings for the CMR + CXA strategy even when this relationship was modified. The fact that the invasive strategies were modeled in this study is certainly a limitation and the 3 different strategies should be assessed in future prospective randomized cost-effectiveness trials. We believe that this study still yields useful results as it is based on real-world data in a patient population of low to intermediate disease prevalence.

In patients with confirmed ischemia, the treatment of symptoms might be more aggressive and consequently more costly. This situation, however, would equally affect costs in the CMR + CXA and CXA + FFR arm. The knowledge of anatomical stenoses could also lead to more aggressive treatment of symptoms and higher costs in the invasive groups. These potential mechanisms could not be taken into account in the current study and could cause underestimations of costs for the invasive strategies.

The registry structure did not allow collecting data to ensure that patients received optimum medical treatment before revascularizations as is recommended by guidelines [5–7, 9, 31] which could influence the outcome. As the outcomes of the CMR + CXA and CXA + FFR strategies were assumed to match, this aspect would not affect the difference of calculated costs for the two strategies. Also, in a recent cost-effectiveness model, for CMR (followed by invasive CXA) and for invasive CXA (including FFR) the quality-adjusted life years gained varied by only 0.08 % (for both, the UK and US systems), while costs varied by 4.7 % and 9.2 %, respectively, in favor of CMR [32].

In general, assigning costs to the various procedures and to hospital stays is a demanding task as some tariff systems are heterogeneous and in addition, differences between geographical regions within a system also exist. This fact has to be considered when interpreting the study results. Costs for FFR were not coded in all tariff systems and therefore, the costs for FFR were calculated as the difference between two tariff positions (for Germany, UK, and Switzerland) or by estimating costs for material [8] and physician payment [33] (for the US). A low reimbursement of this FFR position could disadvantage the CXA-only approach. However, the break-even analysis indicates, that a 6–12 fold increase in the FFR reimbursement would be required to match the costs of the CXA-only strategy.

The CMR + CXA strategy was not compared with other non-invasive imaging stress tests. This aspect warrants testing in future studies.

## Conclusions

A CMR + CXA-guided strategy to manage patients with suspected CAD is less costly than an invasive CXA + FFR strategy when applied in a real-world patient population of low to intermediate prevalence of disease and when assuming same outcomes for the strategies. This finding was observed for the German, UK, Swiss, and US health care systems. However, in the subgroup of patients with typical angina, cost savings were only minimal to moderate. The costs of tests, but also the patient characteristics represent important factors that determine the cost-effectiveness of various work-up strategies. These findings warrant further confirmation in prospective cost-effectiveness trials.

## Additional files

Additional file 1: The stenosis-FFR relationship. (DOC 38 kb)

Additional file 2: Sources of cost calculations. (DOC 74 kb)

Additional file 3: Complictions during CMR. (DOC 37 kb)

Additional file 4: Sensitivity analysis. (DOC 687 kb)

## Abbreviations

CAD: Coronary artery disease
CMR: Cardiovascular magnetic resonance
CXA: X-ray coronary angiography
FFR: Fractional flow reserve
PCI: Percutaneous coronary intervention
CABG: Coronary artery bypass grafting
European CMR registry: European Cardiovascular Magnetic Resonance registry
MACE: Major adverse cardiac events
SCD: sudden cardiac death
MI: Myocardial infarction
nf-MI: non-fatal MI
ab SCD: aborted SCD
