# Supplementary material for: Cost-minimization analysis of three decision strategies for cardiac revascularization: results of the “suspected CAD” cohort of the european cardiovascular magnetic resonance registry
Source: J Cardiovasc Magn Reson. 2016 Jan 11;18:3. doi: 10.1186/s12968-015-0222-1 (PMC4709988; doi:10.1186/s12968-015-0222-1)
Supplement: Supplementary file 1 — The stenosis-FFR relationship. (DOC 38 kb) [file 12968_2015_222_MOESM1_ESM.doc]

**Short Title: Cost evaluation of coronary artery disease management.** K. Moschetti et al.

**Appendix A**

From the literature the relationship between FFR and coronary artery diameter stenosis is known [1] and given as sensitivity, specificity, and accuracy for ≥50% stenoses to detect an FFR ≤0.80. If we assume that the patients with revascularizations are represented by an FFR ≤0.80, the portion of FFR≤0.80 in the current population is 6.2% (=FFR_pos_).

Definitions: Sensitivity (TP / (TP + FN), Specificity (TN / (FP + TN), Accuracy ((TP + TN) / n), where n is the total sample size. Furthermore, TP (=coronary stenosis ≥50% and FFR ≤0.80), TN (=coronary stenosis <50% and FFR >0.80), PF (=coronary stenosis ≥50% and FFR >0.80), FN (=coronary stenosis <50% and FFR ≤0.80), and FFR_pos_ (=TP + FN). The number of patients with a stenosis ≥50% in this population with defined sensitivity, specificity, and accuracy is given by TP + PF and can be expressed as a percentage of the total sample n.

**TP = n . FFR_pos_ . sensitivity**

**FP = [(1 – specificity) . ((n . accuracy) – TP)] / specificity**

**TP + PF in percent of n = (TP + PF) / n . 100%**

According Toth et al. [1] in 4086 vessels the sensitivity, specificity, and accuracy for coronary stenosis ≥50% to detect an FFR ≤0.80 is: 61.2%, 66.9%, and 64.0%, respectively. In the registry population, FFR_pos_ equals 6.2%. With these numbers, TP and FP is calculated as follows:

TP = 4086 **.** 0.062 **.** 0.612; yielding TP = 155

FP = [(1-0.669) **.** ((4086 **.** 0.64) – 155)] / 0.669; yielding FP = 1217

TP + PF (in % n) = (155 + 1217) / 4086 **.** 100%; yielding 33.5%

Accordingly, the stenosis – FFR relationship of the population studied by Toth et al. when applied to the current registry population, yields 33.5% of the registry population with a coronary artery stenosis ≥50%, and thus, this represents the proportion of patients that undergoes FFR testing.

**Reference:**

1. Toth, G., M. Hamilos, S. Pyxaras, F. Mangiacapra, O. Nelis, F. De Vroey, L. Di Serafino, O. Muller, C. Van Mieghem, E. Wyffels, G.R. Heyndrickx, J. Bartunek, M. Vanderheyden, E. Barbato, W. Wijns, B. De Bruyne**. Evolving concepts of angiogram: fractional flow reserve discordances in 4000 coronary stenos***e*s*. Eur Heart* J. 2014**;** 35:2831-2838.
