# Supplementary material for: Cost-minimization analysis of three decision strategies for cardiac revascularization: results of the “suspected CAD” cohort of the european cardiovascular magnetic resonance registry
Source: J Cardiovasc Magn Reson. 2016 Jan 11;18:3. doi: 10.1186/s12968-015-0222-1 (PMC4709988; doi:10.1186/s12968-015-0222-1)
Supplement: Supplementary file 2 — Sources of cost calculations. (DOC 74 kb) [file 12968_2015_222_MOESM2_ESM.doc]

**Short Title: Cost evaluation of coronary artery disease management.** K. Moschetti et al.

**Appendix B – Sources of cost calculations** (+ Table B1)

**Germany:** The uniform value scale (Einheitlicher Bewertungsmaßstab) [1] was used for the outpatient procedure costs. The inpatient procedures are coded in the DRG payment system [2]. Regarding the FFR costs the difference of two DRG payments was used, one including a catheterization+FFR and one including the catheterization only. The CMR cost was derived from the pre-inpatient tariff [3] and was adjusted with an inflation correcting factor to get the 2014 costs [4].

**United Kingdom:** The different diagnostic procedures were derived from 3 references: First, the list of procedure codes (OPCS 4.6) [5]; second, the national Health Related Groups grouper [6]; and third, the 2012-2013 reference costs that provide the national average costs for each HRG [7]. For the CMR costs, the tariff of the British Society of Cardiovascular Magnetic resonance (BSCMR) and the British Society of Cardiovascular Imaging (BSCI) [8] was applied after adjustment with an inflation correcting factor to get the 2014 costs [4]. The FFR costs were calculated as the difference between two HRGs payments: one including the FFR and another without it on the basis of an elective inpatient situation.

**Switzerland:** The outpatient and inpatient procedures are coded in the TARMED [9] and the Swiss Diagnosis Related Groups (DRG) [10] payment systems, respectively.

**United States:** The costs of tests were calculated based on the 2014 average national Medicare payments listed in the Current Procedural Terminology codes (CPT) and the assigned Ambulatory Payment Classification (APC) and DRG categories for the outpatient and inpatient situations, respectively [11, 12]. Medicare does not assign costs for FFR. We therefore calculated it by summing up the costs of the material used [13] and the Medicare average national physician payment assigned to the CPT codes of the test [14]. Costs for a cardiologist’s visit in the first year after revascularization were derived from the statistical brief #381 of the Medical Expenditure Panel Survey [15] and were confirmed by [16, 17]. Cardiology visits costs of 2009 were adjusted with an inflation correcting factor to get the 2014 costs [18].

**Table B1.** Unit costs of the tests and procedures in the 4 countries in 2014

Costs for PCI include costs for diagnostic CXA examination; Costs of one year medication include aspirin and clopidogrel for UK, Switzerland, and US; clopidogrel for Germany only (all generic drugs).

|  | **Germany** (€) | **UK** (£) | **Switzerland** (CHF) | **US** (US$) |
| --- | --- | --- | --- | --- |
| **CMR** (outpatient) | 410 | 585 | 1’420 | 515 |
| **CXA** (outpatient) | 626 | 1’240 | 2’567 | 977 |
| **FFR** (outpatient) | 331 | 512 | 1’931 | 888 |
| **PCI** (in-hospital) | 2’968 | 2’704 | 12’565 | 12’459 |
| **CABG** (in-hospital) | 12’887 | 7’261 | 37’829 | 22’261 |
| **One year medication** | 590 | 36 | 493 | 168 |
| **Cardiologist visit** | 105 | - | 624 | 375 |
| **Aborted SCD** | 4’789 | 3’216 | 29’673 | 17’097 |
| **Stroke** | 4’944 | 2’733 | 8’239 | 8’517 |
| **Non-fatal MI (**without rehab) | 4’252 | 3’945 | 15’484 | 12’535 |
| **Non-fatal MI (**with rehab) | 6’752 | - | 24’369 | - |

**References**
