# Supplementary material for: Cost-minimization analysis of three decision strategies for cardiac revascularization: results of the “suspected CAD” cohort of the european cardiovascular magnetic resonance registry
Source: J Cardiovasc Magn Reson. 2016 Jan 11;18:3. doi: 10.1186/s12968-015-0222-1 (PMC4709988; doi:10.1186/s12968-015-0222-1)
Supplement: Supplementary file 3 — Complictions during CMR. (DOC 37 kb) [file 12968_2015_222_MOESM3_ESM.doc]

**Short Title: Cost evaluation of coronary artery disease management.** K. Moschetti et al.

**Appendix C – Complications during CMR examinations** (Table C)

**Table C**. Complications during CMR examinations (n=3’647)

|  | **Total population** | **Atypical chest pain** | **Typical angina** |
| --- | --- | --- | --- |
| **Incidence** | | | |
| **Major complications** | **0.00%** | **0.00%** | **0.00%** |
| **Minor complications** | **9.69%** | **9.00%** | **13.40%** |
| - Allergy to contrast agent | 0.05% | 0.00% | 0.00% |
| - Angina pectoris | 2.63% | 1.74% | 6.01% |
| - Dyspnea | 4.27% | 4.09% | 4.64% |
| - Non-sustained ventricular tachycardia | 0.13% | 0.17% | 0.00% |
| - Paroxysmal atrial fibrillation | 0.60% | 0.62% | 0.69% |
| - Increase in blood pressure | 0.13% | 0.11% | 0.00% |
| - Decrease in blood pressure | 0.21% | 0.34% | 0.17% |
| - Adenosine-induced broncho-spasm | 0.08% | 0.11% | 0.00% |
| - 1-, 2-, 3-degree AV block | 0.21% | 0.34% | 0.00% |
| - Nausea | 0.74% | 0.90% | 0.86% |
| - Local complications | 0.04% | 0.11% | 0.00% |
| - Claustrophobia | 0.60% | 0.50% | 1.03% |
