# Supplementary material for: Cost-minimization analysis of three decision strategies for cardiac revascularization: results of the “suspected CAD” cohort of the european cardiovascular magnetic resonance registry
Source: J Cardiovasc Magn Reson. 2016 Jan 11;18:3. doi: 10.1186/s12968-015-0222-1 (PMC4709988; doi:10.1186/s12968-015-0222-1)
Supplement: Supplementary file 4 — Sensitivity analysis. (DOC 687 kb) [file 12968_2015_222_MOESM4_ESM.doc]

**Short Title: Cost evaluation of coronary artery disease management.** K. Moschetti et al.

**Appendix D – Sensitivity Analyses** (+ Tables D1-D4)

**Table D1.** Cost related to the 3 strategies in Germany (in €, 2014)

**
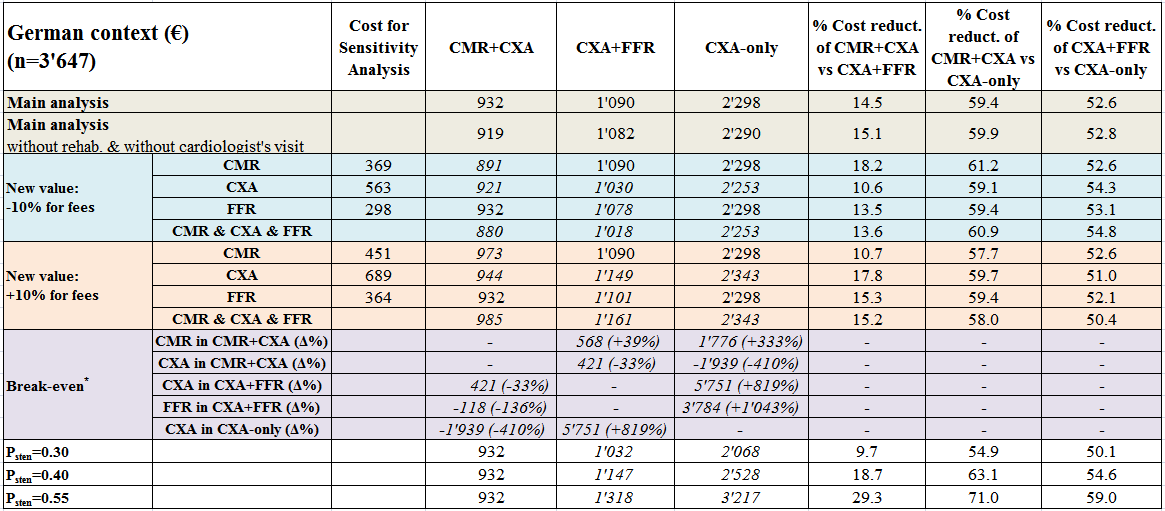
**

**
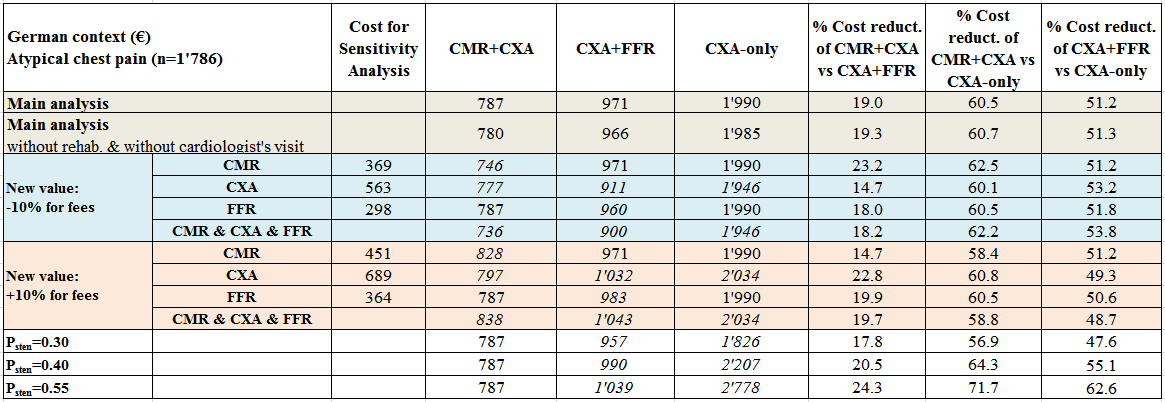
**

**
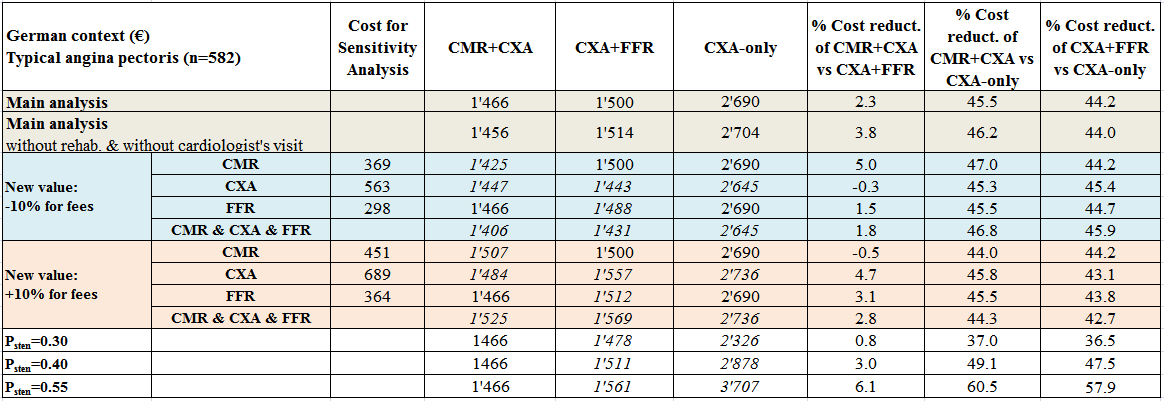
**

| **Table D1:** Sensitivity analyses for Germany include costs for drugs post-revascularization, for rehabilitation after non-fatal MI and for one cardiologist's consultation during the first year after revascularization. Costs with changes in comparison to the main analysis are given in ***italic***. For costs used in the sensitivity analyses, see Table B1 of Appendix B. ^*^Break-even analysis: “CMR in CMR+CXA (Δ%)” e.g. gives the new costs for CMR (and its change in %) to match costs of the CXA+FFR strategy (value in CXA+FFR column) or to match costs of the CXA-only strategy (value in CXA-only column). A negative value indicates, that even no reimbursement would not allow achieving break-even, instead the negative sum must be subtracted from the overall costs to achieve break-even. |
| --- |


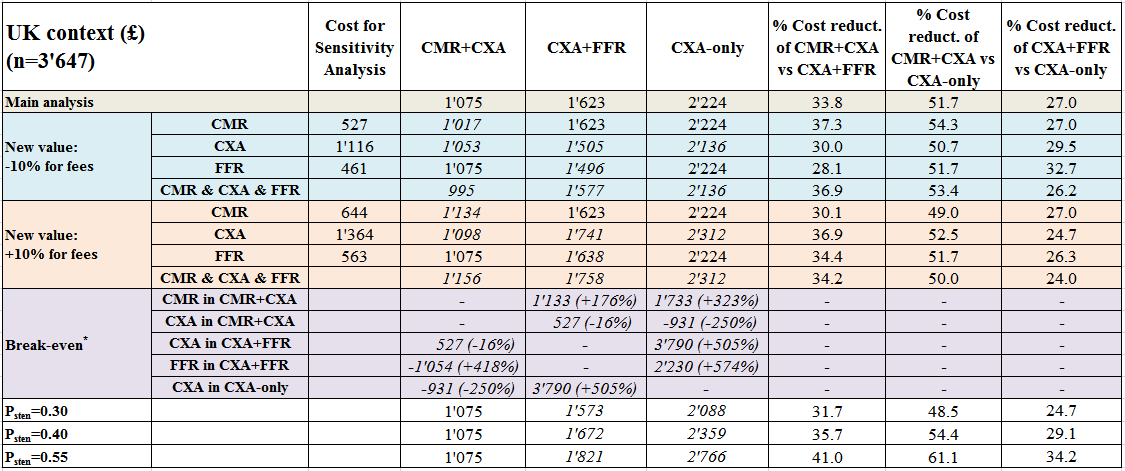
 **Table D2.** Cost related to the 3 strategies in the United Kingdom (in £, 2014)

**
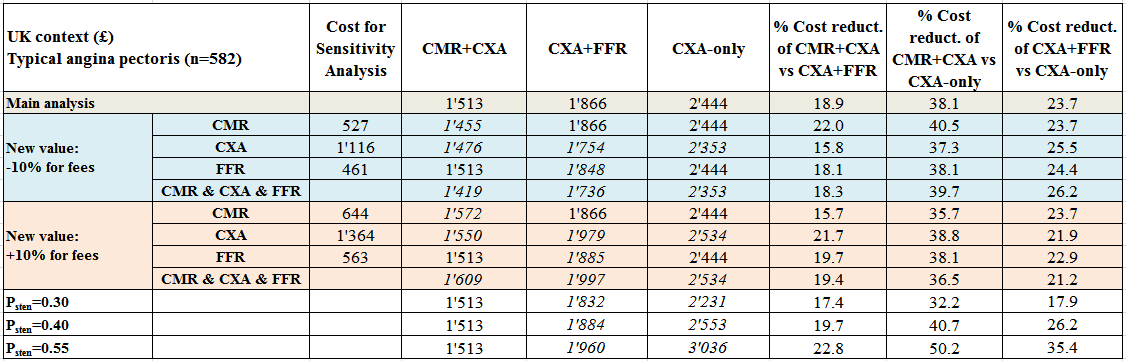

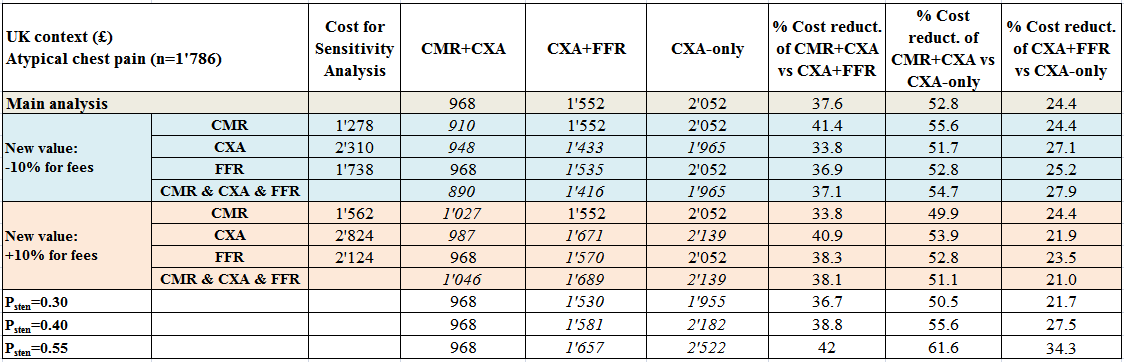
**

| **Table D2:** Sensitivity analysis for the UK include costs for drugs post-revascularization, but are without costs for rehabilitation and without a cardiologist's consultation during the first year after revascularization as patients in UK in general are not assigned to an additional cardiologist’s visit after revascularization and typically they are not undergoing rehabilitation after MI. ^*^See Table D1. |
| --- |


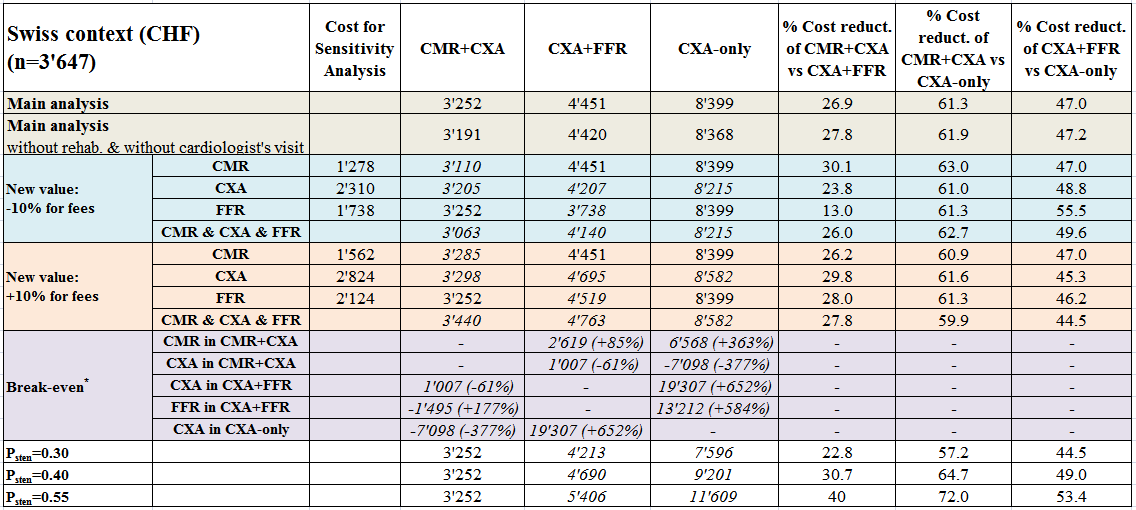
 **Table D3.** Cost related to the 3 strategies in Switzerland (in CHF, 2014)


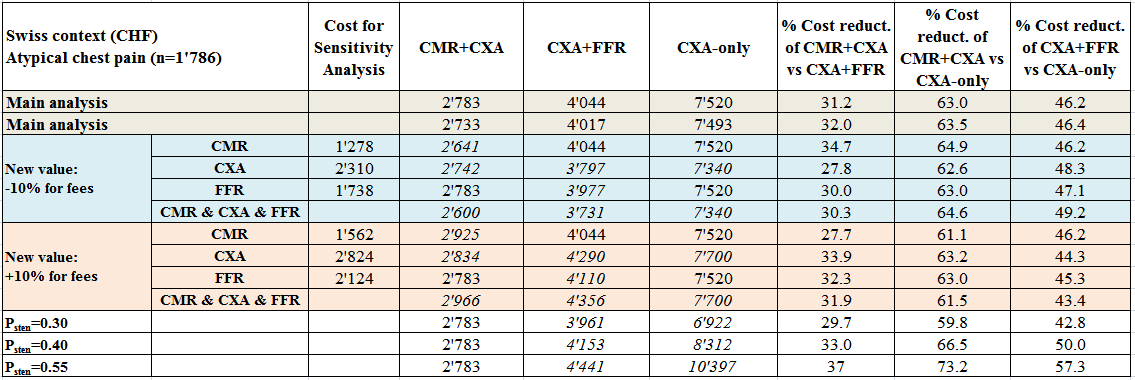

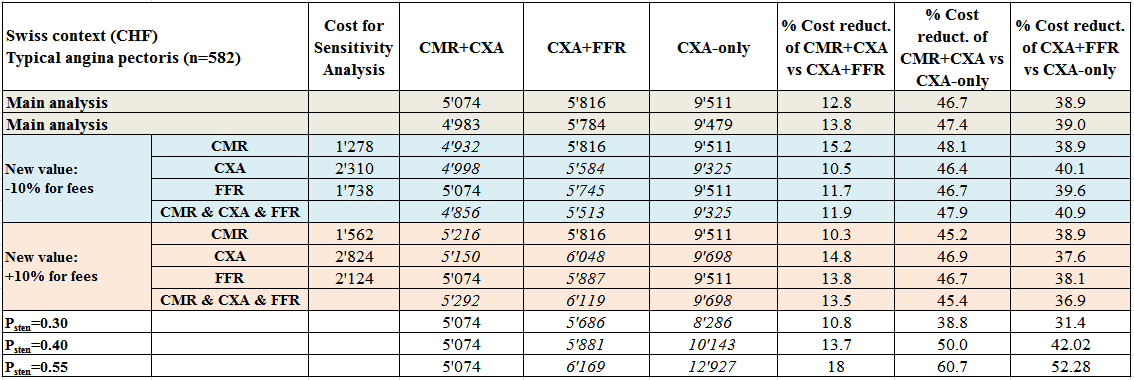
**Table D3:** Sensitivity analyses for Switzerland include costs for drugs post-revascularization, for rehabilitation after non-fatal MI and for a cardiologist's consultation during the first year after

revascularization. ^*^See Table D1.


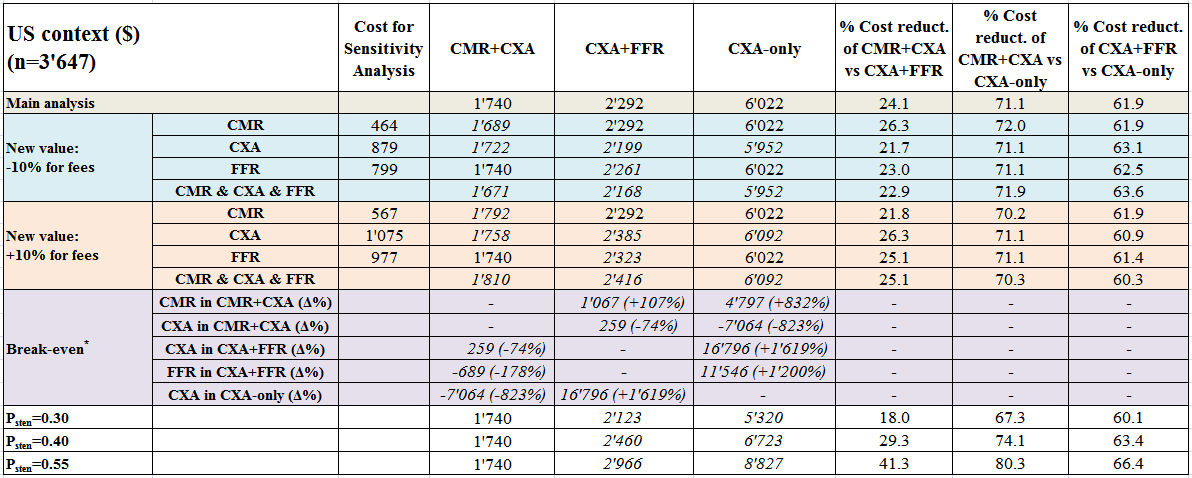
 **Table D4.** Cost related to the 3 strategies in the United States (in US $, 2014)

**
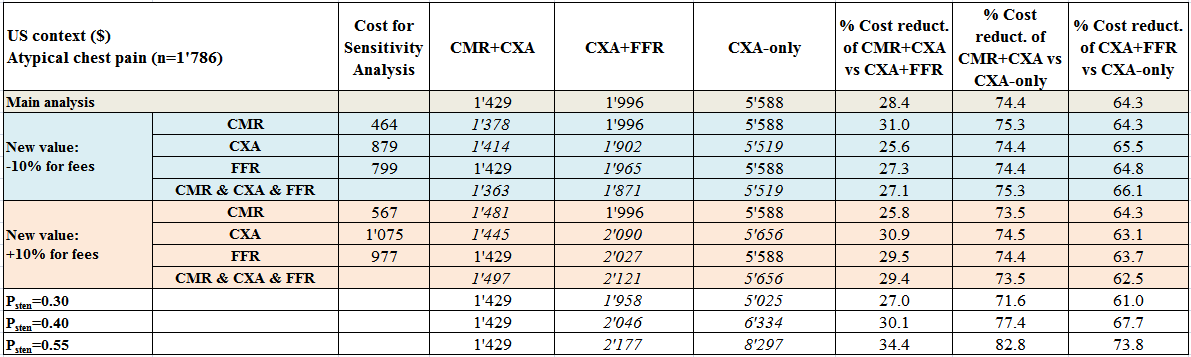
**

**
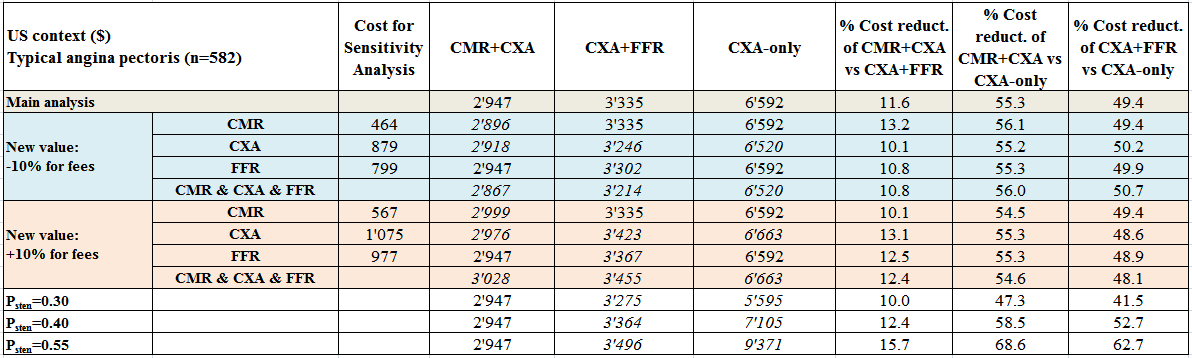
**

**Table D4:** Sensitivity analyses for US include costs for drugs post-revascularization, but are without costs for rehabilitation and without a cardiologist's consultation during the first year after revascularization as patients in US are not assigned to an additional cardiologist’s visit after revascularization on a regular basis and typically they are not undergoing rehabilitation after MI. ^*^See Table D1.
